# A Comparitive Evaluation of the Effect of Sports and Fruit Drinks on the Surface Roughness of Nanofilled Composite and Light Cure GIC-An *In Vitro* Study

**DOI:** 10.5005/jp-journals-10005-1550

**Published:** 2018-10-01

**Authors:** Hemalatha, Priya Nagar

**Affiliations:** 1 Postgraduate Student, Department of Pedodontics and Preventive Dentistry, Krishnadevaraya College of Dental Sciences, Karnataka, India; 2 Professor and HOD, Department of Pedodontics and Preventive Dentistry, Krishnadevaraya College of Dental Sciences, Karnataka, India

**Keywords:** Bindhu jeera fizz, Gatorade, Light cure RMGIC, Nano-filled composite, Tang.

## Abstract

**Context:**

Tooth coloured restorative materials are commonly used for restorations in children and adolescents, who are major consumers of soft drinks. Under acidic conditions, restorative materials suffer degradation over time, which can be predicted by changes in the surface roughness.

**Aim:**

Compare the effect of acidic drinks Gatorade, Tang, Bindhu Jeera Fizz, and 10% sucrose solution (control group) on nano filled composite and light cure RMGIC and test the time dependent change by immersing them in these drinks ever day 8th hourly and examining them on the 10th, 20th and 60th day.

**Methodology:**

Fourty specimens of each material was made and divided equally in 4 groups, group 1 (gatorade), group 2 (Tang), group 3 (Bindhu Jeera Fizz), group 4 (10% sucrose). Each specimen was immersed every 8th hourly daily for 60 days and the surface roughness was assessed on the 0,10th, 20th and 6th day using a with a 3-D optical profilometer.

**Results:**

The surface roughness increased progressively with time with maximum average roughness value (Ra) value was seen on the 60th day in both the materials irrespective of any acidic drink. The highest value of roughness was seen by Group 2 containing Tang (p < 0.001), followed by Bindhu Jeera Fizz (p < 0.001) and Gatorade (p < 0.001) and the least being the 10% Sucrose (control group) (p < 0.001).

**Conclusion:**

The maximum change in surface roughness was associated with light cure RMGIC as compared to Nano-filled composite, mostly due to the low mechanical strength and low wear resistance of glass ionomer restorations making it less durable. Hence nano-filled composite proved to be superior then RMGIC, but with longer exposure to acidic drinks the Ra value increased significantly, hence the consumption of these acidic drinks should be limited.

**How to cite this article:** Hemalatha, Nagar P. A Comparitive Evaluation of the Effect of Sports and Fruit Drinks on the Surface Roughness of nano-filled composite and light cure GIC-An *In vitro* Study. Int J Clin Pediatr Dent. 2018;11(5):417-424.

## INTRODUCTION

Restorative dentistry materials mainly run on two principles which is functional results and aesthetic outcomes. The idlest environment to test the behavior of these restorative materials properties in the mouth; hence they are required to have long-term durability.

Currently, fluoride-releasing aesthetic restorative has been used extensively to counteract caries formation. However, the color stability of these restorative materials has been a challenge to dentistry, as the oral cavity has a dynamic environment.^1^

Glass ionomer cement, as we know releases fluoride ions into underlying dentine which is very effective for treating erosive lesions and further provides the ability to form chemical bonds to the enamel and dentin. However, they are prone to fracture and exhibits low wear resistance.^2,3^

Resin-modified glass ionomer cement was then introduced which claimed to have improved the mechanical properties of glass ionomer cement hence frequently used as occlusal restorations. However, they have a higher incidence of degradation in comparison to resin composite and amalgam.^2^

Composite resins are among the most periodically used aesthetic restorative material in general dental practice, but due to discoloration properties, they remain to be a major problem in long-term clinical studies. Color stability is one of the key factors when selecting composite resin materials for esthetic restorations. Moreover, color stability and discoloration is a major tool to measure the outcome and rate the success and failure of composite resin restorations in dental practices.

Majority of the children and adolescents are major consumers of soft drinks, hence its best to analyze tooth-colored restorative materials on them. Restorative materials suffer degradation over time, especially during an acidic environment, which is very evident by the surface roughness observed due to degradation. This acidic environment is contributed by the oral bacteria which ferment carbohydrates and produce acid, which further dissolves tooth enamel during the dental caries process.^4^

Ismail et al. conducted and reported a study among 9 to 29-year-old to portray the strong association between caries experience and soft drink consumption. Furthermore, caries presence in the upper anterior teeth of 2-year-old was correlated with sugary snacks, particularly sweetened beverages.

Futuristic changes have occurred in the beverages consumed; their manner and role in the diet have changed over time. This leads to detrimental consequences of decreased intakes of calcium and increased rates of childhood obesity. However, the implications of changes in beverage consumption can have for dental caries is not much recognized.^5^

On the contrary a study of competitive athletes, caries experience was not associated with sports drink consumption. Furthermore, the manner in which beverages are consumed (daytime *vs.* nocturnal feedings, snack *vs* meal, prolonged sipping *vs.* quick drinking) probably might influence the disease process, but is extremely difficult to assess in a community setting.^6^

Many current studies showed that significant color changes occur when the composite resins are exposed to dietary colorants and chemical dyes and further if the composites are not fully polymerized. Compositions of the resin matrix affect water sorption, solubility, hydrophilicity, and microstructures of the composites, which may dictate the long-term color stability of the composite resin restorations.^3^

Teeth will remain intact even if sugared juices are frequently taken if proper oral hygiene is maintained and fluoride is supplied frequently, Emslie ^7^ conducted a study in Sudan, stating that sugar consumption is expected to have risen since, due to the economic boost. Depending on individual frequency intake of sugar or sweetened items may eventually provide a feasible plan for the prevention of caries and emphasize the importance of conducting oral health promotion programmes focusing on dietary habits control.

In this present study to assess the surface roughness, we are using the 3D optical profilometer in this study. Comparing profilometer, Atomic Force Microscope (AFM) and Rugosimeter, AFM is used for the qualitative measure and mostly accurate with a much micro size object, and Rugosimeter gives a 2-dimensional representation of the surface roughness and needs a highly shiny surface for measuring accurate results.

Nano filled composites are the newer composites with Nano filler particles with reduced polymerization shrinkage and better surface characteristics and Light cure resin modified glass Ionomer cement are an advanced version of conventional glass Ionomer cement with reduced operator time and faster setting, making it easy to use in pediatric dentistry. Therefore this present study aims to compare the effect of acidic drinks on the surface roughness of nano-filled composite and light cure resin-modified glass-ionomer cement.

## METHODOLOGY

Eighty specimens, of nano-filled composite and light resin modified GIC and were further divided into four subgroups with 10 samples in each subgroup in both Nano-filled composite and Light cure resin-modified GIC as by Random sampling method.

### Group A-Nano Filled Composite

Subgroup

A_1_ Composite in gatorade

A_2_ Composite in Tang

A_3_ Composite in Jeera fizz

A_4_ Compositein 10% sucrose

### Group B-Resin Modified Gic

SUBGROUP

B_1_ Resin-modified GIC in gatorade

B_2_ Resin-modified GIC in tang

B_3_ Resin-modified GIC in Jeera fizz

B_4_ Resin-modified GIC in 10% sucrose

### Specimen Preparation

With the help of a cylindrical aluminum mold with an internal diameter of 5 mm and depth of 2 mm, 40 specimens of each material were prepared, each mold was coated with Vaseline for easy retrieval of the specimens. To get a uniform flat polymerized surface with no bubbles after curing, the top and bottom surfaces were covered with polyester matrix strips (Mylar Strips) and a thin rigid glass slide, with the help of finger pressure excess material was removed on pressing on the glass slide. The material was polymerized using a light emitting diode (LED) light curing unit through the glass slide and polyester matrix strip for 20 seconds. To ensure uniform curing the light probe tip was placed perpendicular to and in contact with the glass slide, such to standardized the distance between the light source and material at 1 mm according to the thickness of the glass slide. All light cured specimens were stored in distilled water in a lightproof container for 24 hours at 37^o^ C to ensure complete polymerization.

### Surface Roughness Testing (Baseline)

Surface roughness testing was done with each restorative material, 40 composite and 40 Light cures RMGIC were randomly divided into two groups. Surface roughness was measured using an optical profilometer.

In this study, each specimen was placed on the platform of the optical profilometer with the test surface facing the optical lens. The mean arithmetic roughness (Ra) was used to assess surface changes. The high value of Ra indicates a rough surface, while a low value will indicate a smooth surface. The mean value of each group was recorded as a baseline roughness measurement (control). The specimens were then stored in individual containers in 20 mL of deionized water at 37^o^ C for 24 hours to allow aging of the sample.

Each sub-group containing 10 samples each was immersed for five minutes three times daily (every 8 hours) which represents the medium frequency of intake of acidic drinks. Before and after immersion in acidic drinks the specimen was rinsed with deionized water, specimens, when not exposed to acidic drinks, were stored in deionized water at 37^o^ C. The juices were refreshed after every immersion for all the drinks.

### Surface Roughness Testing

For all the specimens the surface roughness testing was done on the 10 days (Ist period), 20 days (IInd period), and 60 days (IIIrd period).

## RESULTS

The Surface roughness of each sample was measured with the help of an optical profilometer, on the first day, 10th day, 20th day and 60th day. The results were tabulated and statistically analyzed. Intergroup comparison was done between two groups using student t-test (two-tailed, independent) analysis, followed by intra-group comparison was done using student t-test (two-tailed, dependent) analysis.

### Study Design

An evaluation comparative study.

### Comparison of Surface Roughness in Two Groups on10th day (Table 1 and Graph 1)

The mean surface roughness of was highest in Group 2 containing TANG in both Nano filled composites (Ra-7.76 ±1.98) and RMGIC {6.65±1.18) (p = 0.165). Remaining intergroup comparisons between group A and group B showed a significant difference using student t-test (two-tailed, independent) (p < 0.001).

### Comparison of Surface Roughness in Two Groups on 20th day (Table 2 and Graph 2)

The mean surface roughness of was highest in group 2 containing TANG in nano-filled composites (Ra-13.78 ± 1.95), and it was slightly higher in group 3 containing Bindhu Jeera Fizz in light RMGIC (13.96 ± 0.71) (p = 0.801). Remaining intergroup comparisons between groups A and B showed a significant difference using student t-test (two-tailed, independent) (p < 0.001).

**Table 1:** **Table 1:** Comparison of surface roughness in two groups on10th day

		*Surface roughness @ 10th day*					
*Subgroups*		*Group A*		*Group B*		*p-value*	
1		2.66 ± 1.14		4.83 ± 0.89		< 0.001**	
2		7.76 ± 1.98		6.65 ± 1.18		0.165	
3		3.96 ± 1.32		6.43 ± 0.72		< 0.001**	
4		2.80 ± 0.68		4.16 ± 0.82		0.001**	

**Table 2:** **Table 2:** Comparison of surface roughness in two groups on 20th day

		*Surface roughness @ 20th day*					
*Subgroups*		*Group A*		*Group B*		*p-value*	
1		8.22 ± 1.04		7.37 ± 1.12		0.114	
2		13.78 ± 1.95		13.96 ± 0.71		0.801	
3		10.32 ± 1.43		14.81± 2.32		< 0.001**	
4		4.02 ± 0.67		4.54 ± 0.82		0.159	

**Graph 1:**
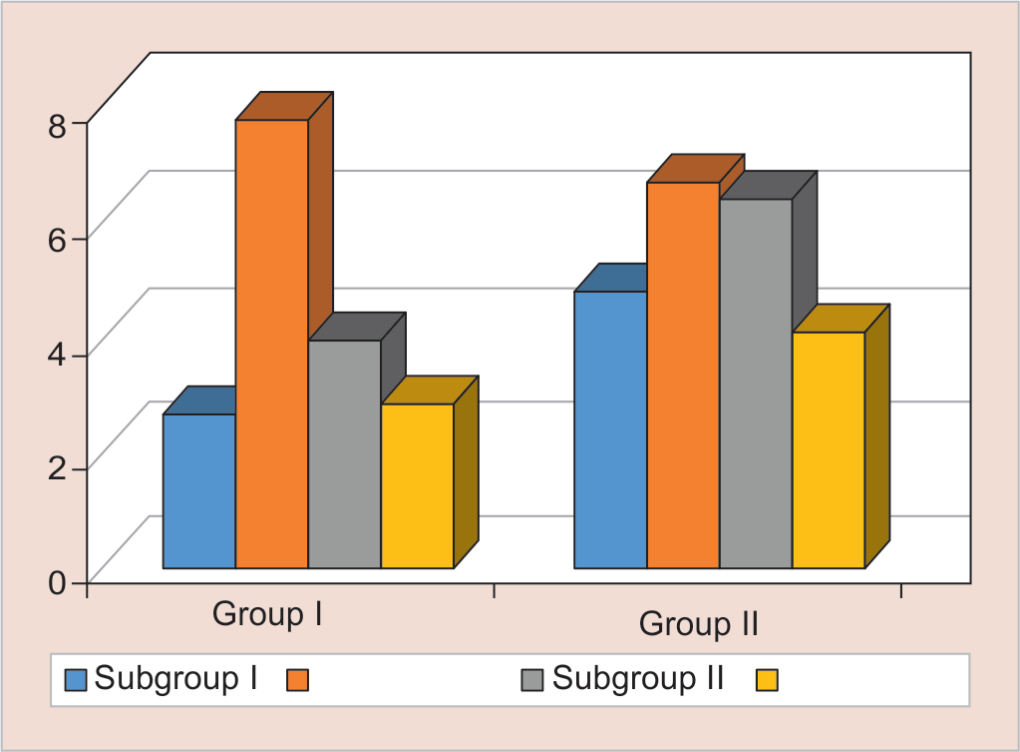
Comparison of surface roughness in two groups on10th day

**Graph 2:**
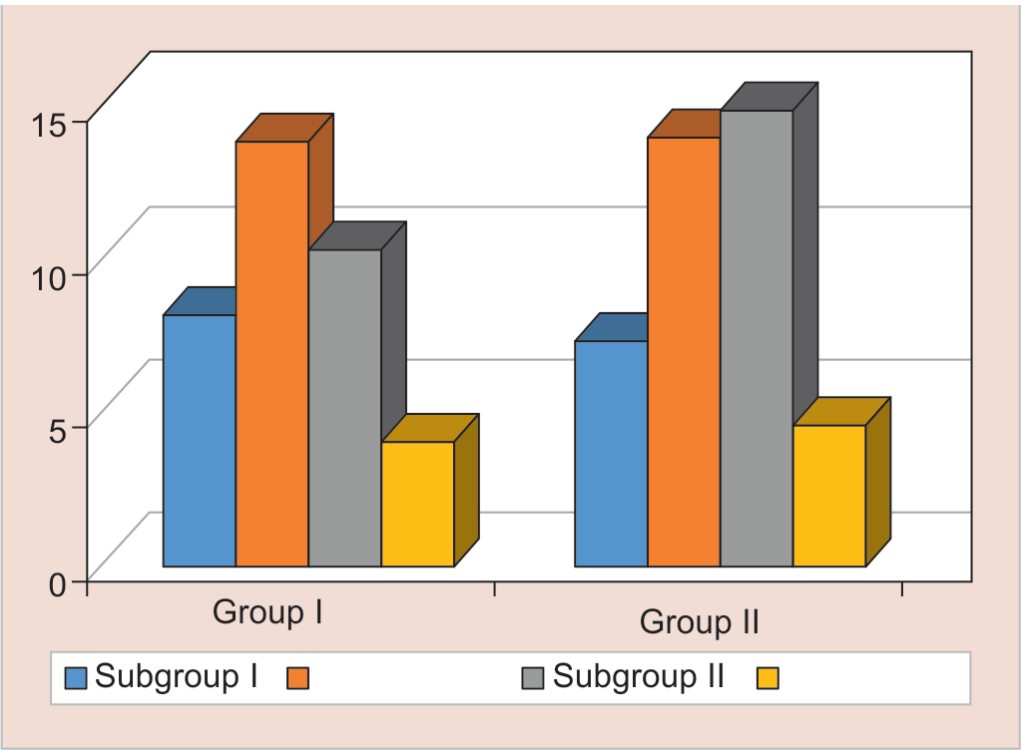
Comparison of surface roughness in two groups on 20th day

### Comparison of Surface Roughness in Two Groups on 60th day (Table 3 and Graph 3)

The mean surface roughness of was highest in group 2 containing Tang in nano-filled composites (Ra-22.00 ± 1.27), and it was slightly higher in Group 3 containing Bindhu Jeera Fizz in light RMGIC (Ra-16.41 ± 0.68) (p < 0.001). Remaining intergroup comparisons between group A and group B showed a significant difference using student t-test (two-tailed, independent) (p < 0.001).

### Group A: Assessment of Surface Roughness from the 0th day to 10th day, 20th day and 60th day

A1: Assessment of surface roughness (Graph 4 and Table 4)

The mean surface roughness of Nano Filled Composite immersed in Gatorade was measured on the 0, 10th, 20th and 60th day using One Way ANOVA and there was a significant increase in the Ra value with highest value observed on the 60th day (Ra-16.6) (p < 0.001).

A2: Assessment of surface roughness (Graph 5 and Table 5)

The mean surface roughness of nano-filledcomposite immersed in Tang was measured on the 0, 10th, 20th and 60th day using One Way ANOVA and there was a significant increase in the Ra value with highest value observed on the 60th day (Ra-22) (p < 0.001).

A3: Assessment of surface roughness (Graph 6 and Table 6)

The mean surface roughness of nano-filled Composite immersed in Bindhu Jeera fizz was measured on the 0, 10th, 20th and 60th day using One Way ANOVA and there was a significant increase in the Ra value with highest value observed on the 60th day (11.48) (p < 0.001).

A4: Assessment of surface roughness (Graph 7 and Table 7)

The mean surface roughness of nano-filled Composite immersed in 10% Sucrose (control group) was measured on the 0, 10th, 20th and 60th day using One Way ANOVA and there was a significant increase in the Ra value with the highest value observed on the 60th day (8.68) (p < 0.001).

**Table 3:** **Table 3:** Comparison of surface roughness in two groups on 60th day

*Subgroups*		*Surface roughness @ 60th day*				*p-value*	
		*Group A*		*Group B*			
1		18.07 ± 0.95		13.96 ± 1.02		< 0.001**	
2		22.00 ±1.27		16.41 ± 0.68		< 0.001**	
3		11.48 ± 0.82		20.18 ± 0.69		< 0.001**	
4		8.68 ± 0.79		7.82 ± 0.67		0.025*	

**Table 4:** **Table 4:** A1-Assessment of surface roughness

*A1*		*Min-Max*		*Mean ± SD*		*Difference*		*t-value*		*p-value*	
0 day		0.23-0.34		0.30 ± 0.04		–		–		–	
10th day		1.25-5.01		2.66 ± 1.14		2.362		6.234		< 0.001**	
20th day		6.80--9.40		8.22 ± 1.04		7.922		22.863		< 0.001**	
60th day		16.60-19.30		18.07 ±0.95		17.766		56.273		< 0.001**	

**Graph 3:**
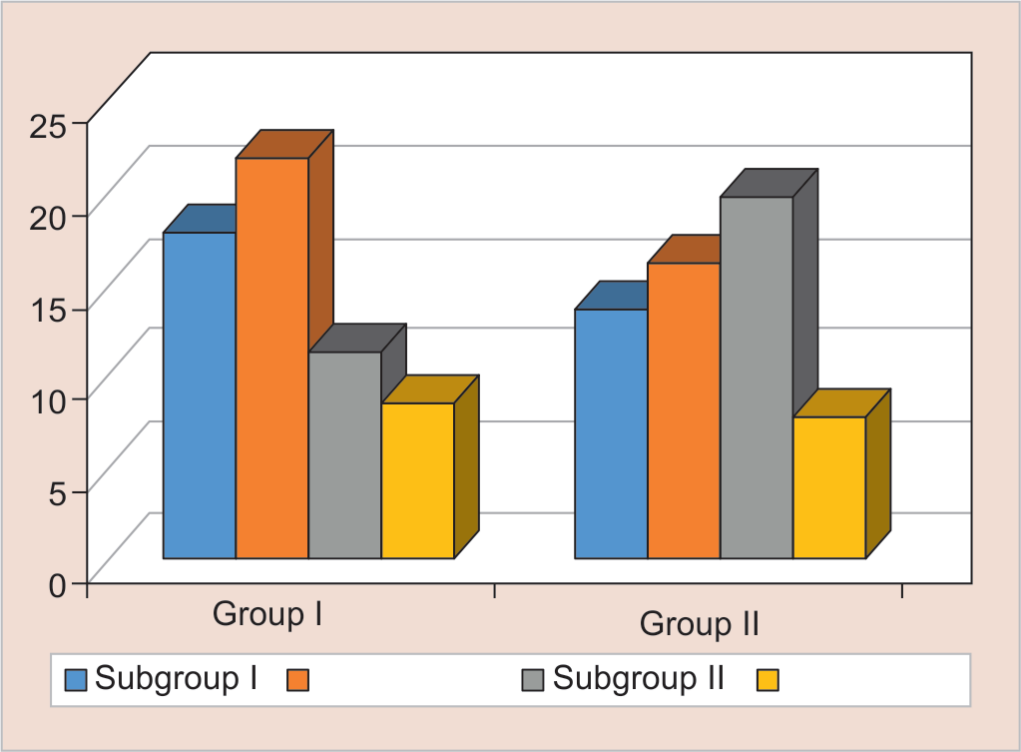
Comparison of surface roughness in two groups on 60th day

**Graph 4:**
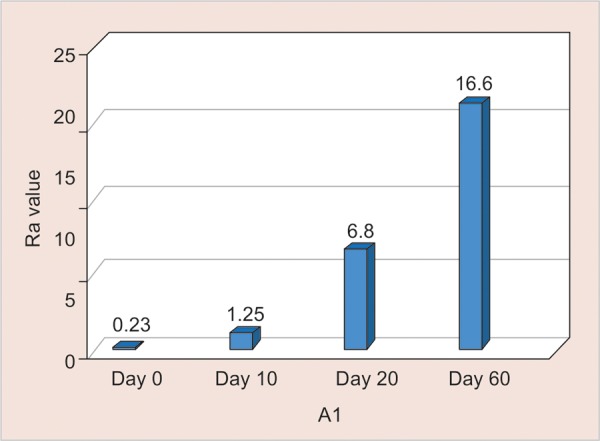
Assessment of surface roughness of A1

### Group B: Assessment of Surface Roughness from the 0th day at 10th day, 20th day and 60th day

B1: Assessment of Surface Roughness (Graph 8 and Table 8)

The mean surface roughness of Light Cured Resin Modified GIC immersed in Gatorade was measured on the 0, 10th, 20th and 60th day using One Way ANOVA and there was a significant increase in the Ra value with highest value observed on the 60th day (13.96) (p <0.001).

B2: Assessment of Surface Roughness (Graph 9 and Table 9)

The mean surface roughness of Light Cure Resin Modified GIC immersed in Tang was measured on the 0, 10th, 20th and 60th day using One Way ANOVA and it showed a significant increase in the Ra value on 10th, 20th and 60th day. (p < 0.001) and the highest value of 16.41 seen on the 60^th^ day.

B3: Assessment of Surface Roughness (Graph 10 and Table 10)

The mean surface roughness of Light Cure Resin Modified GIC immersed in Bindhu Jeera Fizz was measured on the 0, 10th, 20th and 60th day using ONE WAY ANOVA and there was a significant increase in the Ra value with highest value observed on the 60^th^ day (20.18) (< 0.001).

B4: Assessment of Surface Roughness (Graph 11 and Table 11)

The mean surface roughness of light cure resin modified GIC immersed in 10% Sucrose solution (control group) was measured on the 0, 10th, 20th and 60th day using One Way ANOVA and there was a significant increase in the Ra value with highest value observed on the 60th day (7.82) (p < 0.001).

## DISCUSSION

Restorative materials are exposed to changes in temperature and acidic-base conditions from food and drinks in the oral cavity. Surface characteristics such as roughness determine the clinical quality and performance of restorative materials during restorative procedures.^2,8^

Surface roughness and irregularities make the restorations more prone to dental plaque accumulation, further leading to gingival irritation, and reduce the aesthetics and the longevity of the restorative materials. A major source of bacteria in the oral cavity is the bacterial accumulation on the surfaces of restorative materials which may further lead to secondary caries formation. Hence properties help us determine the choice of restorative material to be used.^2,3,9^

The pH of the solution profoundly influences the surface value it’s exposed to as the pH of the environment decreases, the roughness value increases because in acid solutions and prolonged exposure of these glass ionomer materials to acids would result in higher Ra values.^10^

**Table 5:** **Table 5:** A2-Assessment of surface roughness

*A2*		*Min-Max*		*Mean ± SD*		*Difference*		*t-value*		*p-value*	
0 day		0.23-0.34		0.30 ± 0.03		–		–		–	
10th day		2.91-9.91		7.76 ± 1.98		7.463		11.314		< 0.001**	
20th day		11.70-17.70		13.78 ± 1.95		13.479		20.761		< 0.001**	
60th day		20.40-23.90		22.00 ± 1.27		21.701		50.849		< 0.001**	

**Table 6:** **Table 6:** A3-Assessment of surface roughness

*A3*		*Min-Max*		*Mean ± SD*		*Difference*		*t-value*		*p-value*	
0 day		0.23-0.34		0.30 ± 0.04		–		–		–	
10th day		2.00-6.01		3.96 ± 1.32		3.660		8.192		< 0.001**	
20th day		8.70-12.80		10.32 ± 1.43		10.027		20.731		< 0.001**	
60th day		10.50-12.90		11.48 ± 0.82		11.184		40.588		< 0.001**	

**Graph 5:**
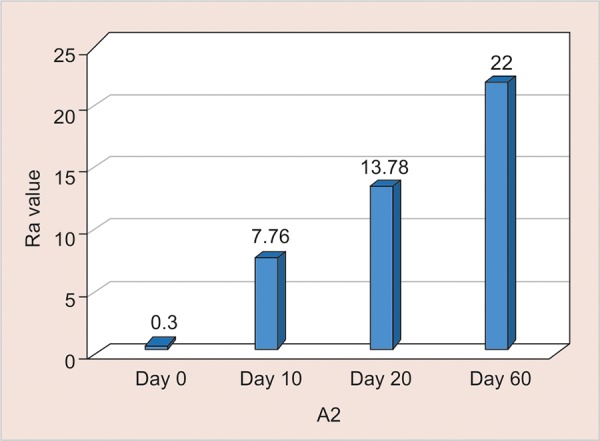
Assessment of surface roughness of A2

**Graph 6:**
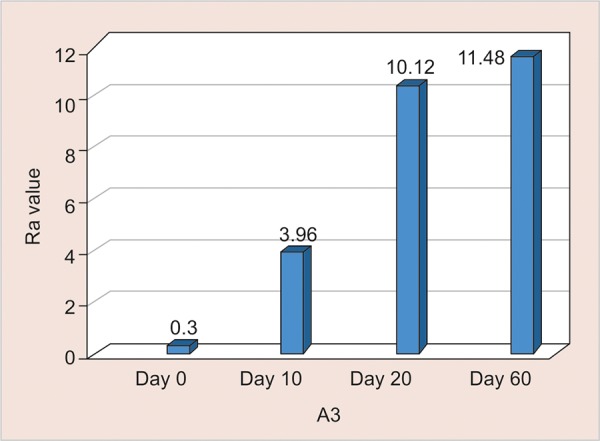
Assessment of surface roughness of A3

**Table 7:** **Table 7:** A4-Assessment of surface roughness

*A4*		*Min-Max*		*Mean ± SD*		*Difference*		*t-value*		*p-value*	
0 day		0.23-0.35		0.30 ± 0.04		–		–		–	
10th day		1.52-3.54		2.80 ± 0.68		2.496		11.002		< 0.001**	
20th day		3.10-5.00		4.02 ± 0.67		3.718		17.112		< 0.001**	
60th day		7.90-10.30		8.68 ± 0.79		8.373		31.073		< 0.001**	

**Table 8:** **Table 8:** B1-Assessment of surface roughness

*B1*		*Min-Max*		*Mean ± SD*		*Difference*		*t-value*		*p-value*	
0 day		1.70-3.20		2.47 ± 0.51		–		–		–	
10th day		3.60-6.60		4.83 ± 0.89		2.359		5.657		< 0.001**	
20th day		5.10-8.98		7.37 ± 1.12		4.904		12.073		< 0.001**	
60th day		12.20-15.50		13.96 ± 1.02		11.497		35.941		< 0.001**	

**Graph 7:**
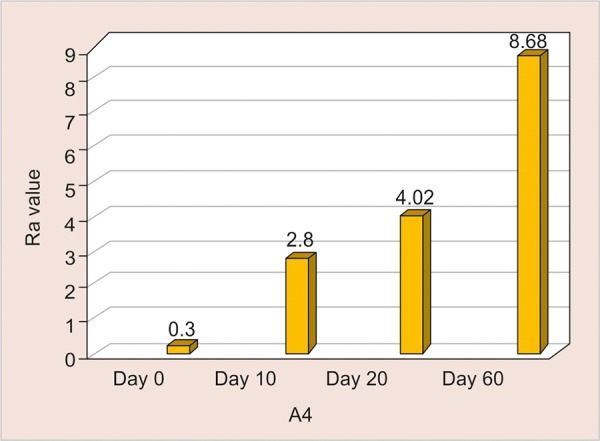
Assessment of surface roughness of A4

**Graph 8:**
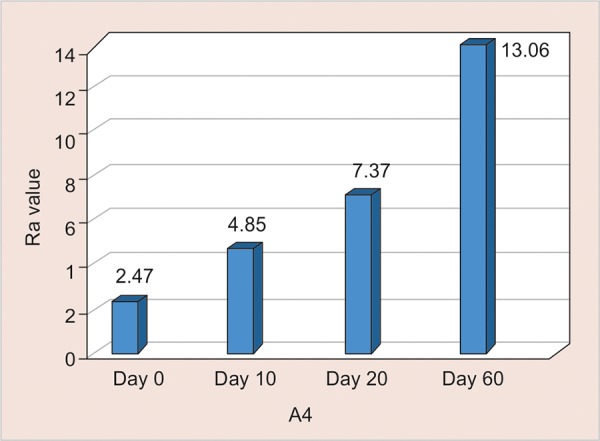
Assessment of surface roughness of B1

**Table 9:** **Table 9:** B2-Assessment of surface roughness

*B2*		*Min-Max*		*Mean ± SD*		*Difference*		*t-value*		*p-value*	
0 day		1.70-4.20		2.76 ± 0.77		–		–		–	
10th day		5.25-9.25		6.65 ± 1.18		3.890		8.327		< 0.001**	
20th day		13.00-15.10		13.96 ± 0.71		11.200		43.836		< 0.001**	
60th day		15.32-17.20		16.41 ± 0.68		13.652		52.415		< 0.001**	

**Table 10:** **Table 10:** B3-Assessment of surface roughness

*B3*		*Min-Max*		*Mean ± SD*		*Difference*		*t-value*		*p-value*	
0 day		1.70-4.20		2.77 ± 0.74		–		–		–	
10th day		5.38-7.38		6.43 ± 0.72		3.668		11.504		< 0.001**	
20th day		12.00-19.10		14.81 ± 2.32		12.044		15.549		< 0.001**	
60th day		18.91-21.10		20.18 ± 0.69		17.410		52.591		< 0.001**	

**Graph 9:**
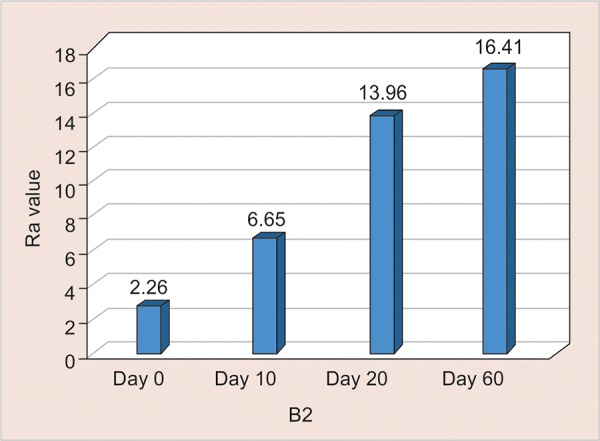
Assessment of surface roughness of B2

**Graph 10:**
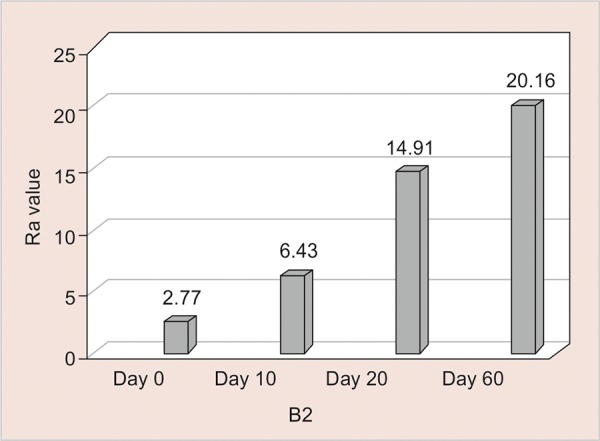
Assessment of surface roughness of B3

The widespread use of resin-based restorative materials requires them to be resistant to the harsh conditions of the oral environment. Nano-filled composite has been known to show more resistance to degradation because of its lower surface roughness compared with micro hybrid resin due to smaller particle size; hence the wear resistance of Nano filled composites will be higher due to its unique properties of greater homogeneity and lesser particles on surface.^11^

In the oral environment, due to its acidic medium, there are both dissolution of elements and erosion of the non-soluble components of the restorative material. Machado et al. (2007) ^12^ also stated that the acidity effect of carbonated beverages is mainly due to phosphoric acid, may produce high levels of tooth erosion and harmful impact on tooth-colored restorative materials due to its chelating properties. In the present study, all the drinks tested were acidic in nature , Gatorade (pH-2.92), TANG (pH-2.7) Bindhu Jeera Fizz (pH-2.5) and 10% Sucrose (pH-5.5), hence 10% sucrose solution is used s the control due to least acidic pH compared to all.

**Table 11:** **Table 11:** B4-Assessment of surface roughness

*B4*		*Min-Max*		*Mean ± SD*		*Difference*		*t-value*		*p-value*	
0 day		1.70-4.20		2.94 ± 0.92		–		–		–	
10th day		3.21-5.64		4.16 ± 0.82		1.217		2.534		0.035*	
20th day		3.44-6.11		4.54 ± 0.82		1.598		3.108		0.014*	
60th day		7.23-8.91		7.82 ± 0.67		4.877		11.161		< 0.001**	

**Graph 11:**
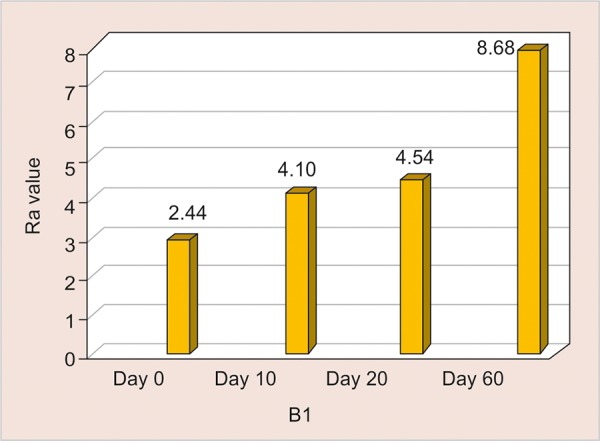
Assessment of surface roughness of B4

In the present study light cure, RMGIC and nano-filled composite is treated with four kinds of acidic drinks, namely Gatorade sports drink, Tang fruit flavoured juice, Bindhu Jeera Fizz and 10% Sucrose solution (control group), for 10, 20 and 60 days. The surface roughness increased progressively with time with maximum Ra value seen at the 60th day in both the materials irrespective of any acidic drink. (Graphs 1 to 3 and Tables 1 to 3). Lamis A Al-Taie^13^ stated exposure to soft drinks for 60 days significantly affects the surface integrity of resin composite materials measured. Composite resins with larger filler particle size, and lower filler volume are probably more prone to degradation in acidic environments. Prakki^14^ stated that pH affects reaction rates which leads to the formation of more carboxylic groups through catalysis by hydrolysis of ester groups present in the resin matrix, that could further lower the pH inside the polymeric matrix. Narsimha et al.^15^ added chronic exposure to acidic media invariably affected both the marginal integrity and microhardness of RMGIC, and the concluded that the marginal integrity and surface deterioration of the material studied is directly proportional to the frequency of exposure to acidic drinks.

Group 2 containing Tang saw the highest value of roughness, (p < 0.001).followed by Bindhu Jeera fizz (p < 0.001) and Gatorade (p < 0.001).and the least being the 10% Sucrose (Control group) (p < 0.001) (Tables 1 to 3 and Graphs 1 to 3). This was observed due to the acidic pH of Tang being more then Gatorade. (Graphs 4, 5, 8 and 9, and Tables 4, 5, 8 and 9). Maganur et al.16 stated that the patient’s fruit beverage consumption habit might affect the longevity of the restorations, cause the erosive effect of both aerated drink and fresh fruit juice caused surface roughness on both flowable composite and RMGIC restorative materials. Hence eventually affect the longevity of the restorations.

The maximum change in surface roughness was associated with Vitremer (Light Cure RMGIC) as compared to 3M Filtek (nano-filled composite), mostly due to the low mechanical strength and low wear resistance of glass ionomer restorations making it less durable (Tables 1 to 3 and Graphs 1 to 3). Nazish et al.^17^ stated that Resin composite (Filtek Z350) materials are more impervious to acidic degradation than resin-modified glass ionomer cement (Vitremer), when submerged in acidic agents proved to tested have an equal effect on the surface hardness of restorative materials. Deionized water had is inert on either restorative material. Hence Nano-filled composite proved to be superior then RMGIC, but with longer exposure to acidic drinks, the Ra value increased significantly.

In the present study, there was a significant increase in the surface roughness of both nano filled composites and light cure RMGIC when immersed in Gatorade which demonstrated the highest Ra value on the 60th day. Similar results obtained in a study by Al-Samadani et al.^18^ who compared different energy drink, concluding that high surface roughness was observed after 6 months. This shows that energy drinks over longer duration cause more erosive effects than other beverages.

Nazish et al.^19^ contradicting the above results by stating that the Surface microhardness of composite resin materials were notably decreased when immersed in sports drinks on day 1, but insignificant reduction was seen after the 14 month evaluation period.

This explains the least change in the surface Roughness in both the materials when immersed in Gatorade from day 0, 10th, 20th and 60th day (Graphs 4 and 8 and Tables 4 and 8) when compared with the other groups.

The least amount of surface roughness was observed in Nano filled Composites compared to Light Cure RMGIC, when they were immersed in 10% Sucrose solution (control group). The least amount of surface roughness was exhibited by both the groups immersed in 10% Sucrose solution (control group) in comparison to Gatorade, Tang and Bindhu Jeera fizz (Graphs 7 and 11 and Tables 7 and 11).
